# Algicidal Activity and Microcystin-LR Destruction by a Novel Strain *Penicillium* sp. GF3 Isolated from the Gulf of Finland (Baltic Sea)

**DOI:** 10.3390/toxins15100607

**Published:** 2023-10-10

**Authors:** Irina Kuzikova, Tatyana Zaytseva, Ekaterina Chernova, Anna Sazanova, Andrey Sharov, Nadezda Medvedeva

**Affiliations:** 1 Scientific Research Centre for Ecological Safety, St. Petersburg Federal Research Center, Russian Academy of Sciences, St. Petersburg 197110, Russia; zaytseva.62@list.ru (T.Z.); s3561389@yandex.ru (E.C.); sharov_an@mail.ru (A.S.); ngmedvedeva@gmail.com (N.M.); 2 All-Russia Research Institute for Agricultural Microbiology (ARRIAM), St. Petersburg 196608, Russia; anna_sazanova@mail.ru; 3 Papanin Institute for Biology of Inland Waters, Russian Academy of Sciences, Borok 152742, Russia

**Keywords:** harmful algae blooms, microcystin-LR, fungi, algicidal activity, biotransformation, toxicity, glutathione, catalase

## Abstract

The present article focuses on a strain of ascomycete GF3 isolated from a water sample taken in the Gulf of Finland. Based on phylogenetic analysis data, the isolate was identified as *Penicillium* sp. GF3. The fungus GF3 demonstrates algicidal activity towards cyanobacteria (98–100%). The algicidal effect on green algae did not exceed 50%. The isolate GF3 exhibits an indirect attack mode by releasing metabolites with algicidal and/or lytic activity into the environment. Moreover, the strain *Penicillium* sp. GF3 is able to degrade MC-LR. After 72 h of GF3 cultivation, the MC-LR content was reduced by 34.1% and 26.7% at initial 0.1 μg/mL and 0.45 μg/mL concentrations, respectively. The high stress resistance of the GF3 to toxic MC-LR is provided by a 1.5-fold activation of catalase activity and a change in the reduced glutathione content. Additionally, during the MC-LR biotransformation, a MC-LR-GSH conjugate and linearized MC-LR were identified. The linearized MC-LR in the presence of fungi capable of degrading MCs was revealed for the first time. Using *Daphnia magna* as a bioindicator, it was shown that the MC-LR biotransformation led to the formation of less toxic intermediates. The toxicity of the fungal filtrate is reduced by five times compared to the abiotic control. Our findings enhance the understanding of the role that ascomycete fungi have as potential bioagents for cyanoHABs to control and detoxify water bodies.

## 1. Introduction

Climate change and eutrophication of water bodies lead to the massive development of cyanobacteria in natural water bodies, which is recognized as a global environmental issue [1]. The increasing intensity of “blooms” in water bodies raises concerns due to the fact that many of the cyanobacteria species that cause “bloom” can form potent toxins [2]. The most common and well-studied cyanotoxins are microcystins (MCs). MCs present in cyanobacterial cells are released into the environment after lysis [3]. Among all representative MCs, microcystin-LR (MC-LR) is the most dangerous in view of its pronounced hepatotoxicity [4]. The mechanism of MC-LR toxicity is considered as a multistep process that includes the inhibition of protein Ser/Thr phosphatases PP1/PP2A and the formation of reactive oxygen species [5,6]. MC-LR negatively affects different mammalian body systems, including the nervous [7], digestive [8], respiratory [9], etc. Exposure to MC-LR can lead to the occurrence and progression of various types of cancer [10]. In 2010, the International Agency for Research on Cancer classified MC-LR as a Group 2B carcinogen [11]. The toxic effects of MC-LR extend to many aquatic species, from invertebrates to mollusks, fish [12], and plants [13].

Currently, the problem of harmful algae blooms (HAB_s_) is extremely relevant for the northwest of Russia. HABs are frequently observed in various water areas of the northwest of Russia, including the Gulf of Finland. In the eastern part of the Gulf of Finland, toxic species of cyanobacteria producing MCs (*Microcystis aeruginosa*, *Dolichospermum lemmermannii*, *Planktothrix agardhii*) have been identified. Eleven different structural variants of MCs were detected [14].

To address the issue of toxic “blooms” and the degradation of MCs, many physical (adsorption, photolysis, sonication, etc.) [15,16], chemical (algicides) [17], and biological (viruses, bacteria, fungi, plants) [18,19,20,21] methods and other technologies have been developed and tested. Among these, biological methods are considered the most economical and environmentally friendly way to combat the mass development of cyanobacteria, as they promote further decomposition of microcystins [22]. However, most biological studies were focused mainly on the search for the bacteria with the algicidal properties, and the ability to degrade MCs [23]. In contrast, the potential of fungi to control the mass development of cyanobacteria and reduce the content of MCs has been studied to a lesser extent. Currently, a number of fungi belonging to basidiomycetes, ascomycetes, and zygomycetes has been known to inhibit cyanobacterial growth and lyse their cells, while only six species are capable to degrade MCs [24]. The study by Mohamed et al. [18] demonstrated that the ascomycete strain *Trichoderma citrinoviride* kku-0955 has the ability not only to inhibit the growth of *Microcystis aeruginosa*, but also to completely degrade microcystins produced by this cyanobacterium within 5 days of incubation with living fungal mycelia.

Like bacteria, fungi can degrade MCs through enzymes into less toxic compounds and use them as carbon sources [18]. In addition, fungi induce a decrease in the toxicity of cyanobacteria, probably through the release of the metabolites that inhibit the expressions of the microcystin synthase genes and block the synthesis of MC in cyanobacterial cells [25,26]. Han et al. [26] found that the *Penicillium chrysogenum* strain inhibited the growth of *M. aeruginosa* by secreting extracellular substances (penicillin V and penicillin G). *P. chrysogenum* also reduced the transcriptional abundances of genes *mcy*A, *mcy*B, and *mcy*D involved in microcystin synthesis, and the expressions of genes *psa*B and *rbc*L involved in photosynthesis. 

The study aimed (i) to isolate and identify a new ascomycete strain with the algicidal properties and the ability to degrade MC-LR; (ii) to study the spectrum of its algicidal activity; (iii) to survey the process and mechanisms of transformation of MC-LR by the isolated fungus; and (iv) to evaluate fungal responses to oxidative stress caused by MC-LR.

## 2. Results and Discussion

The open water area in the easternmost part of the Baltic Sea, in particular the Russian part of the Gulf of Finland, has been usually less prone to harmful water blooms compared to the coastal waters of the Gulf of Finland (Kurortny District) [14]. In a water sample collected near the Gogland Island in the Gulf of Finland in June 2018, potentially toxigenic species of cyanobacteria *Aphanizomenon flos-aquae*, *Dolichospermum lemmermannii*, and *Nodularia spumigena* were identified. In the water sample, five structural variants of MCs were detected (total content 442.7 ng/L), with the most toxic representative MC-LR (306.4 ng/L) making the largest contribution (Table 1). *Dolichospermum lemmermannii* was the most likely MCs producer.

### 2.1. Isolation and Identification of the GF3 Strain

The strain of *Penicillium* sp. GF3 was isolated from the water sample taken during the active vegetation of cyanobacteria in the Gulf of Finland. Colonies of the GF3 strain showed limited growth on Czapek’s agar, reaching a diameter of 20 ± 1 mm after 14 days at a temperature of 25 °C. The surface of the colonies was velvety; the sporulation was pale bluish-grayish; the exudate was abundant; the reverse of the colonies ranged from yellow to amber; and the pigment slowly diffused into the agar. 

**Figure 1:**
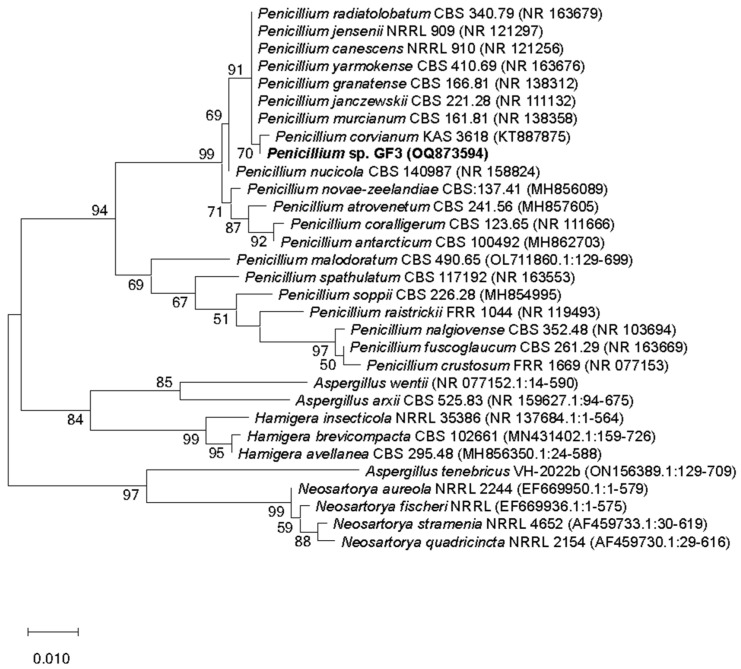
Phylogenetic tree for the strain *Penicillium* sp. GF3 and the closest strain types built on the basis of ITS-region sequences using the maximum likelihood method. Only bootstrap values higher than 50% are presented. Strain *Penicillium* sp. GF3 is in bold.

The level of sequence similarity of the ITS strain *Penicillium* sp. GF3 was 99.82% with the closest type strains of the genera *P. murcianum* CBS 161.81, *P. corvianum* KAS 3618, *P. canescens* NRRL 910, *P. radiatolobatum* CBS 340.79, and *P. jensenii* NRRL 909. In the phylogenetic tree built on the basis of the sequences of the ITS region, the GF3 strain entered the main cluster with the type strains of the genus *Penicillium* at a support level of 91% (Figure 1). Within this cluster, the strain GF3 formed a subgroup with the strain type *P. corvianum* KAS 3618; however, its species affiliation could not be determined due to the low level of support (70%). Based on the results obtained, the isolated strain was identified as *Penicillium* sp. GF3.

Fungi of the genus *Penicillium*, which include more than 350 species, are widespread both in terrestrial and aquatic ecosystems, and can enter into contact with various other representatives of the biota. It has been established that fungi of the genus *Penicillium* are able to inhibit the growth and lyse cells of *Microcystis aeruginosa* [26,27]. Metabolites of the *P. chrysogenum* strain were found to manifest an inhibitory effect on the expressions of genes that control photosynthesis and MC synthesis in *M. aeruginosa* [26]. Information on the ability of fungi of the genus *Penicillium* to degrade MCs is currently lacking.

### 2.2. The Spectrum of the Algicidal Activity of Strain GF3

**Figure 2:**
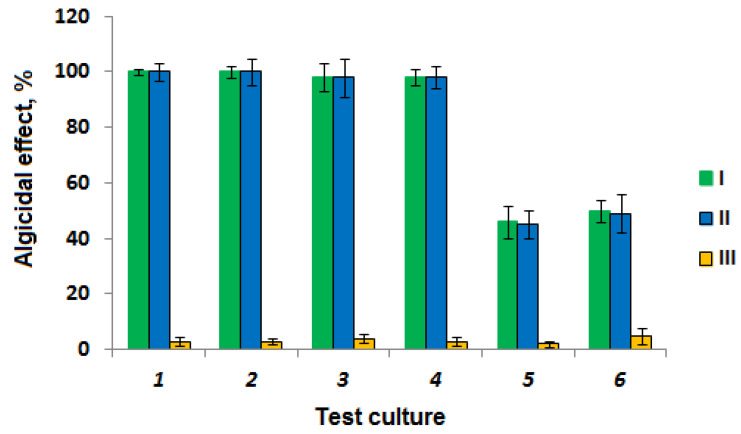
Algicidal effect of strain GF3 on the test cultures for 24 h: 1—*Microcystis aeruginosa* Kütz. (CALU 973); 2—*Planktothrix agardhii* Gom Anagnostidis et Komarek (CALU 1113); 3—*Aphanizomenon flos-aquae* (Linnaeus) Ralfs ex Bornet and Flahault (CALU 1033); 4—*Anabaena cylindrica* Lemm. (HPDP); 5—*Oocystis parva* W. and G.S. West (CALU 391); 6—*Scenedesmus quadricauda* (Turp.) Breb. (CALU 1248); I—culture liquid; II—filtrate; III—washed mycelium.

The results of the determination of the GF3 strain algicidal activity showed that the culture liquid of the fungus and the GF3 filtrate manifested an algicidal effect on both cyanobacteria and green algae (Figure 2).

The highest sensitivity to the algicidal action of strain GF3 was noted in cyanobacteria. After 24 h, the content of chlorophyll a in the medium with the addition of culture liquid or culture filtrate of the GF3 strain decreased by 98–100% for all tested cyanobacteria. The algicidal effect of the isolated strain on green algae was lower, and did not exceed 50%. It should be noted that the washed mycelium of the fungus did not have an algicidal effect on the studied test cultures (Figure 2). Based on the obtained results, the mechanism of action of the GF3 strain on cyanobacterial and algal cells can be attributed to an indirect effect through the excretion of metabolites with algicidal and/or lytic activity. Similarly, an indirect mechanism of the algicidal action was previously noted in ascomycetes of the genera *Acremonium*, *Emericellopsis*, *Aureobasidium*, *Penicillium*, and *Trichoderma* [18,27,28,29]. These fungi inhibit the growth of cyanobacteria by the action of enzymes, antibiotics, and other unidentified biologically active metabolites released into the cultivation medium. Direct contact lysis of the cyanobacterial cells was revealed only for basidiomycetes of the genera *Irpex*, *Phanerochaete*, and *Trichaptum* [27,30,31].

Han and colleagues reported the algicidal activities of fungi of the genus *Penicillium*. The efficiency of chlorophyll a inhibition in *Microcystis aeruginosa* PCC7806, when co-cultivated with fungi *Penicillium janthinellum* F48, *Penicillium* sp. F06, *Penicillium* sp. F07, and *Penicillium virgatum* F22, ranged from 76.5 to 85.4% after 120 h. However, these strains have not been studied for their ability to degrade MC-LR [27].

### 2.3. MC-LR Removal by the Strain Penicillium sp. GF3

**Figure 3:**
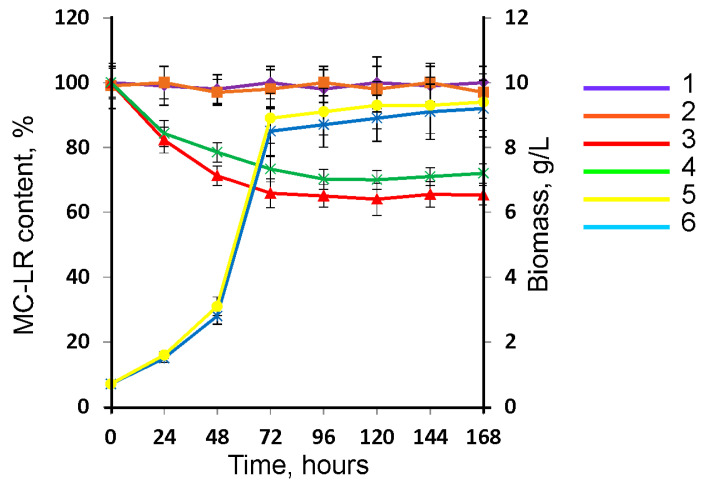
Dependence of the MC-LR content in the native solution and the biomass of the *Penicillium* sp. GF3 on the time: 1—MC-LR content in abiotic control; 2—MC-LR content with inactivated GF3 strain biomass; 3—MC-LR content at initial concentration 0.1 µg/mL MC-LR; 4—MC-LR content at initial concentration 0.45 μg/mL MC-LR; 5—GF3 strain biomass (control); 6—GF3 strain biomass at initial concentration 0.45 μg/mL MC-LR.

The MC-LR removal was studied at the initial concentrations of the MC-LR of 0.1 and 0.45 μg/mL in the medium. Figure 3 data indicates that the decrease in MC-LR concentration occurred due to biological processes, since in the abiotic control, the concentration of MC-LR did not change during the entire cultivation process.

After 72 h of the strain *Penicillium* sp. GF3 cultivation, the content of the raw MC-LR at the concentrations of 0.1 µg/mL and 0.45 µg/mL in the native solution decreased statistically significantly (*p* < 0.05) by 34.1% and 26.7%, respectively. No further removal of the toxin was observed at both concentrations studied. It should be noted that inactivated fungal cells did not adsorb MC-LR. Based on the data obtained, it could be concluded that the removal of MC-LR from the solution occurs due to its biodegradation by the GF3 strain, rather than through sorption.

A comparative analysis of the efficiency of biodegradation of the MC-LR by *Penicillium* sp. GF3 showed that the studied strain is superior to the known MC biodestructor *Mucor hiemalis* EH5 (DSM 14200). Thus, the *M. hiemalis* EH5 strain eliminated 37% of MC-LR from the medium at a concentration of 0.03 μg/mL, which was an order of magnitude lower than in our study, by in the first 24 h; and then there was no decrease in the content of the MC-LR [32].

It should be noted that MC-LR at the studied concentrations up to 0.45 μg/mL does not affect the growth and biomass production of the destructor strain (Figure 3). Similar data on the absence of toxic effects of MC-LR on the growth of fungi were obtained by other researchers. Thus, MC-LR at concentrations up to 1μg/mL did not inhibit the growth of the fungus *Mucor hiemalis* EH5 and the yeast *Saccharomyces cerevisiae* [33,34].

### 2.4. Determination of Lipid Peroxidation and Antioxidant

It is known that MCs can induce oxidative stress in the cells of various living organisms—animals, fish, plants, cyanobacteria, and yeasts [33,35,36,37]. As a result of oxidative stress, lipid peroxidation (LPO) occurs in cells, resulting in damage to cell membrane structures [38]. In addition, LPO products, in particular malondialdehyde (MDA), can have mutagenic activity and block cell division [39].

**Figure 4:**
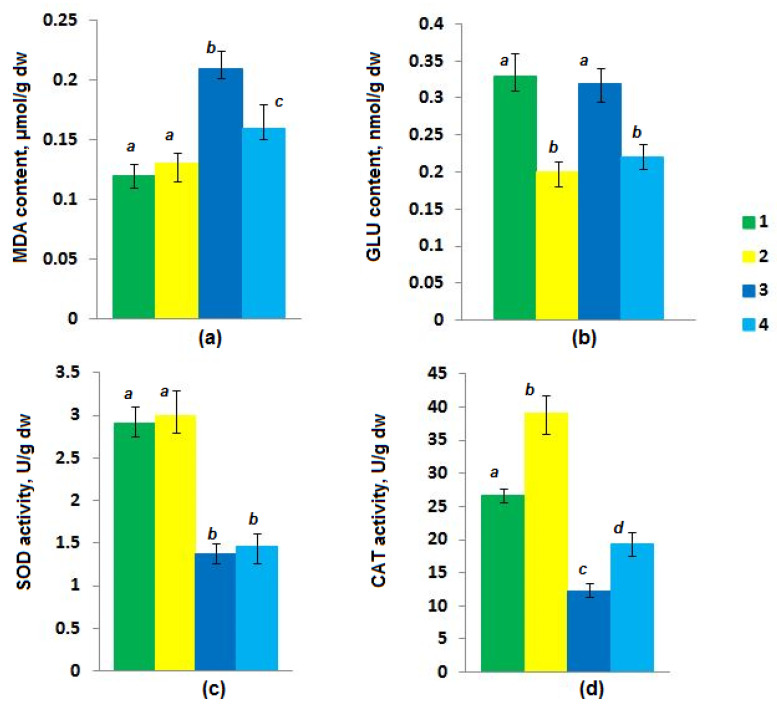
The content of malondialdehyde (**a**), reduced glutathione (**b**), and the activity of superoxide dismutase (**c**) and catalase (**d**) in the cells of the fungus *Penicillium* sp. GF3 during cultivation: 1—control (72 h without MC-LR); 2—0.45 µg/mL MC-LR (72 h); 3—control (120 h without MC-LR); 4—0.45 µg/mL MC-LR (120 h).

MDA, a biological marker of oxidative stress and the main product of lipid peroxidation, was examined in our study. The amount of MDA in the cells of the studied fungus did not significantly differ (*p* > 0.05) from the control after 72 h of the *Penicillium* sp. GF3 cultivation in the presence of MC-LR. However, at 120 h, it was statistically significantly lower at 15% (*p* < 0.05) compared to the control (Figure 4a).

In this research, to elucidate the resistance of strain GF3 to MC-LR, we studied the response of the antioxidant defense system of GF3 strain to the exposure of MC-LR.

The high stress resistance of the strain to the toxic metabolite of cyanobacteria, MC-LR, can be caused by the activation of the synthesis of certain metabolites involved in the protection of cells from oxidative stress induced by toxic substances. The enzymes superoxide dismutase (SOD) and catalase, which facilitate reactions with primary reactive oxygen species, are the key components of the cell defense system against oxidative stress [40].

In our study, the SOD activity in the presence of MC-LR was at the level of control values, and no statistically significant difference (*p* > 0.05) was recorded in *Penicillium* sp. GF3 after 72 and 120 h of incubation (Figure 4c).

In contrast to the SOD activity, strain GF3 exhibited a 46% increase in catalase activity after 72 h of cultivation, which further increased by another 10% at 120 h (Figure 4d). A similar reaction of the enzyme defense systems to oxidative stress (through activation of catalase and superoxide dismutase) induced by MC-LR at concentrations of 0.001 µg/mL and 1 µg/mL was previously recorded in the yeast *Saccharomyces cerevisiae* [33]. Since catalase is one of the main enzymes that protect the cell from the toxic effects of hydrogen peroxide, the authors suggest that the main reactive oxygen species (ROS) induced by MC-LR is hydrogen peroxide [33]. In contrast to the ascomycete strain GF3 and *Saccharomyces cerevisiae*, the activity of catalase, as well as superoxide dismutase, in the zygomycete *Mucor hiemalis* EH5 (DSM 14200) were unchanged under the action of MC-LR (0.001, 0.01, and 0.1 μg/mL) [41].

It has been known that along with enzymatic factors protecting cells from oxidative stress, intracellular glutathione with glutathione-S-transferase as a catalyst is able to conjugate with reactive oxygen species, lipid peroxidation products, xenobiotics, toxins, and/or their metabolites. Glutathione is one of the most abundant thiol compounds in eukaryotic cells. Intracellular glutathione was irreversibly consumed during the conjugation with toxic substances [33,42,43]. 

The obtained data on the content of the reduced glutathione in the *Penicillium* sp. GF3 cells degrading MC-LR are shown in Figure 4b. Thus, the results indicated that during the MC-LR destruction by the GF3 strain, a statistically significant (*p* < 0.05) decrease in the content of reduced glutathione by 39%, compared to the control, was observed in fungal cells after 72 h of cultivation. This decrease persisted after 120 h of cultivation. The revealed low level of reduced glutathione in GF3 cells is likely due to its consumption in the conjugation process with MC-LR.

### 2.5. Determination of MC-LR Biotransformation Products

It is known that the initial step in the biotransformation of MC-LR by higher plants, invertebrates, and mollusks is the formation of the MC-LR-glutathione conjugate (MC-LR-GSH). This reaction occurs between the nucleophilic center in the methyldehydroalanine (Mdha) group of MC-LR and the sulfhydryl group of reduced glutathione, catalyzed by glutathione-S-transferase [44]. It is one of the most common types of toxin modification. MC-LR-GSH is expected to be 3–10 times less toxic to mammals than MC-LR [5].

**Figure 5:**
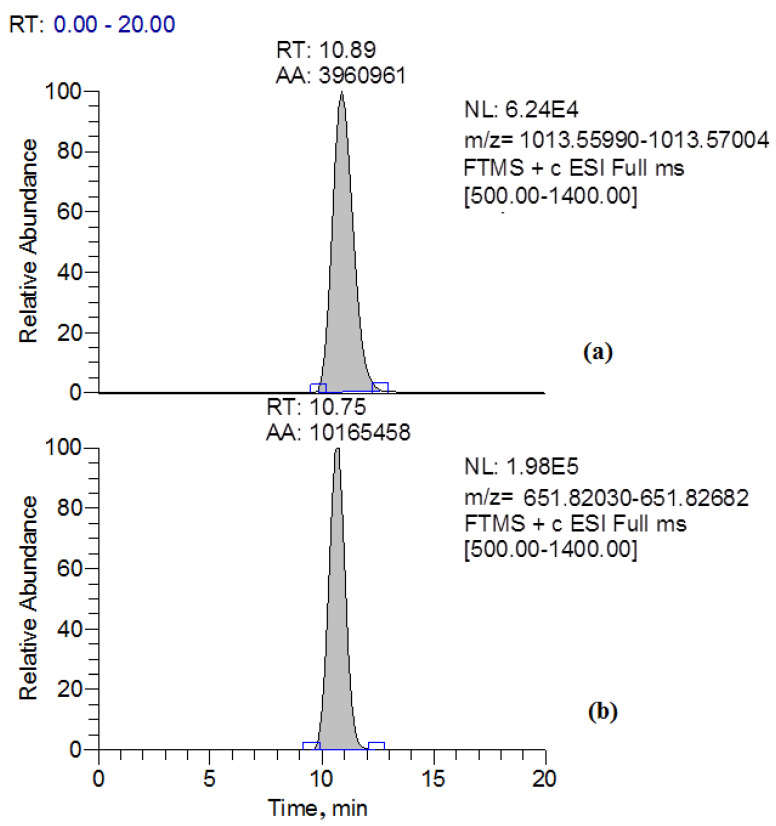
Extracted ion chromatograms of the products of microcystin-LR transformation by *Penicillium* sp. GF3. (**a**)—linearized form of microcystin LR, (**b**)—MC-LR-GSH.

**Figure 6:**
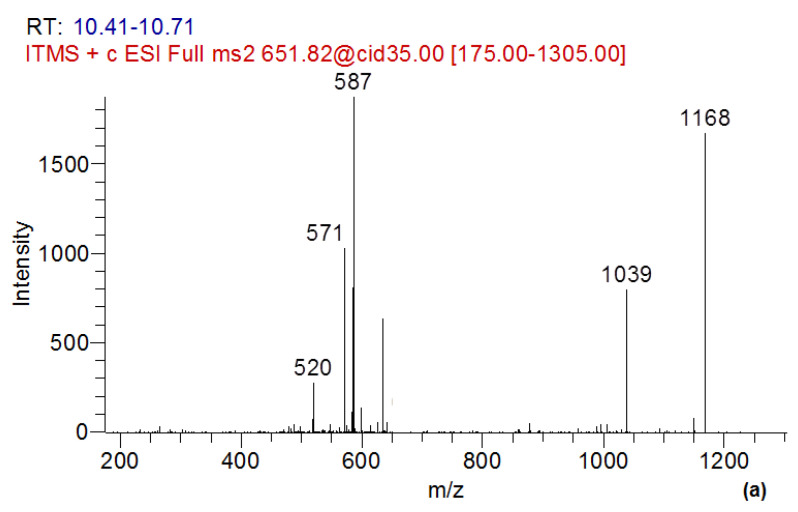

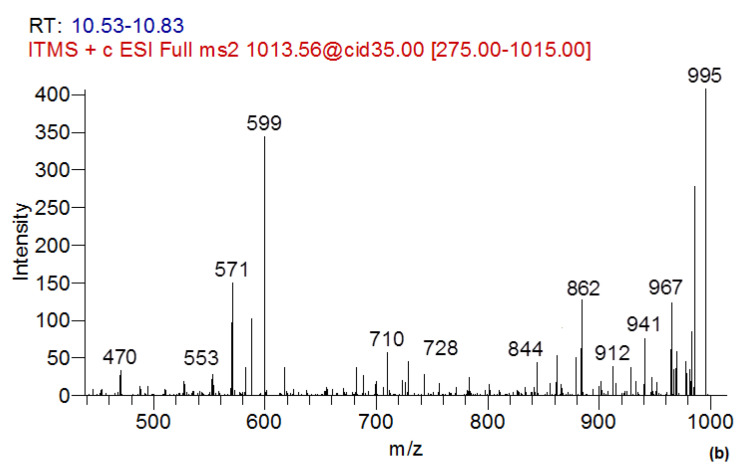
Tandem mass spectra of MC-LR biotransformation products: (**a**) MC-LR-GSH (precursor ion–double charged molecular ion with *m*/*z* 651), (**b**) linearized MC-LR (precursor ion–molecular ion with *m*/*z* 1013).

In the present study, simultaneously with a decrease in the content of MC-LR in the culture liquid of the destructor fungus GF3, two compounds related to the products of transformation of MC-LR were identified. These compounds were MC-LR-GSH, which was identified as a doubly charged ion with *m*/*z* 651.82356, and the linearized form of MC-LR (ADDA-Glu-Mdha-Ala-Leu-Masp-Arg-OH), which has a protonated form with *m*/*z* 1013.56497 (Figure 5 and Figure 6).

The identification of the MC-LR conjugate with glutathione was carried out using the exact mass of the doubly charged protonated molecular ion, as well as according to the data of fragment spectra. The fragment spectrum of the precursor ion with *m*/*z* 651, corresponding to MC-LR-GSH, contained the product ion with *m*/*z* 587 [32] (Figure 6a).

Previously, among the known fungi that destroy MC-LR, the glutathione conjugate MC-LR-GSH was found only in the strain *Mucor hiemalis* EH5 (DSM 14200) [32].

The fragment spectra of the linearized form of MC-LR (Figure 6b) contained a number of characteristic ion products with *m*/*z* 995; 862; 571 [45], and the additional ion products presented in the fragment spectrum of MC-LR with *m*/*z* 844; 728; 710; 599; 571; 553; 470 [46]. It should be noted that the linearized form of MC-LR in fungi destructors was revealed for the first time.

It has been established that the transformation of cyclic MC-LR into a linear one can be carried out by bacteria. The degradation of MC-LR by bacterial cultures is mediated by at least three intracellular hydrolases [45,47]. The MlrA, MlrB, and MlrC genes, which are involved in the cleavage of the microcystin ring structure, were detected with PCR. However, the genes responsible for MC degradation by fungi have not yet been identified.

### 2.6. Evaluation of GF3 Filtrate Toxicity

**Figure 7:**
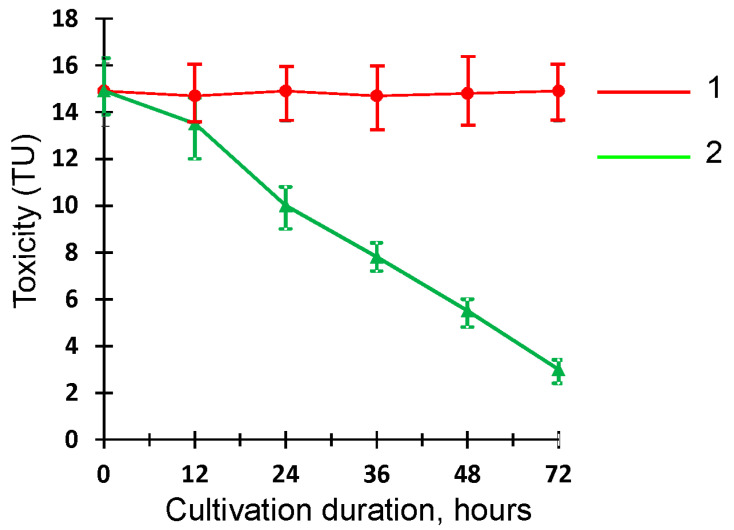
Toxicity of GF3 filtrate against *Daphnia magna* during cultivation with MC-LR: 1—abiotic control; 2—culture filtrate.

To assess the acute toxicity of MC-LR and its metabolic transformation products by *Penicillium* sp. GF3, the Daphtoxkit F bioassay was used. This toxicity bioassay was performed using the freshwater crustacean species *Daphnia magna*, a widely used bioindicator of acute toxicity of various pollutants such as pesticides, dyes, mycotoxins, and other xenobiotics [48]. The results of the toxicological experiment are presented in Figure 7.

The bioassay results showed that the biotransformation of MC-LR by *Penicillium* sp. GF3 leads to the formation of less toxic degradation products compared to the original compound. In the process of the destruction, a statistically significant decrease (*p* < 0.05) in the toxicity (TU) of GF3 filtrate by almost 1.5-fold and 5-fold compared to the abiotic control was observed after 24 and 72 h of cultivation of the strain, respectively. This reduction in toxicity correlated with a decrease in the toxin content in the native solution. On the contrary, no changes in toxicity occurred in the abiotic control throughout the entire period of incubation, suggesting that a decrease in the toxicity of the culture liquid occurred due to biodegradation and/or biotransformation of MC-LR by the *Penicillium* sp. GF3. In addition, it was confirmed that the biotic control represented by the GF3 filtrate without MC-LR had no acute toxicity against *Daphnia magna*.

## 3. Conclusions

Thus, the revealed high potential of the algicidal properties of the strain *Penicillium* sp. GF3, along with its ability to degrade highly toxic MC–LR with the formation of less toxic transformation products, suggests further investigations of its potential for preventing the mass development of cyanobacteria and detoxifying water bodies contaminated with toxic metabolites of cyanobacteria.

## 4. Materials and Methods

### 4.1. Water Sampling

The water sample was collected during the visual observation of the “bloom spot” near Gogland Island in the Gulf of Finland in the eastern part of the Baltic Sea, in June 2018. The sampling point coordinates were 60.04963° N, 27.15025° E. Sampling was carried out according to the previously described procedure [14].

### 4.2. Isolation and Identification of the GF3 Strain

The strain of *Penicillium* sp. GF3 was isolated from a water sample taken in the Gulf of Finland near Gogland Island. The fungus isolation was carried out on a solid Czapek nutrient medium with 2% glucose and streptomycin (100 μg/mL) at 25 °C for 14 days. The strain was identified by cultural and morphological features using standard methods and determinants [49]. Strain identification was also performed using a molecular method for sequencing the ITS region of DNA. Isolation, amplification, and sequencing of fungal genomic DNA were carried out in accordance with the previously described procedure [50]. DNA was isolated using a DiaGen reagent kit (Dia-M, St. Petersburg, Russia) in accordance with the manufacturer’s recommendations. The ITS1-5.8S-ITS2 region was amplified with primers ITS1 (5′-TCCGTAGGTGAACCTGCGG-3′) and ITS4 (5′-TCCTCCGCTTATTGATATGC-3′). The sequencing of the ITS region was performed using an ABI 3500xl genetic analyzer (Applied Biosystems, Waltham, MA, USA). The sequence of the ITS region of the strain *Penicillium* sp. GF3 was compared to the corresponding strain sequences available in the GenBank database using NCBI BLAST analysis: https://www.ncbi.nlm.nih.gov/nuccore/OQ873594 (accessed on 1 May 2023). The phylogenetic tree was constructed using the maximum likelihood method using the MEGA-X software package (v. 10.2) [51]. Evolutionary distances were calculated using the maximum composite likelihood method. A bootstrap analysis with 1000 replicates was performed to evaluate cluster support. The nucleotide sequence of the strain was deposited in the GenBank database under the number OQ873594.

### 4.3. Cultivation of the Strain GF3

The cultivation of the strain was carried out using Czapek’s liquid medium with 2% glucose in the dark on a Certomat BS-1 incubation shaker (Sartorius Stedim Biotech, Göttingen, Germany) at 230 rpm and 25 ± 1 °C for 168 h. As inoculum (10% vol), a 2-day-old culture grown on the above-mentioned medium in the exponential growth phase was used. To obtain the inoculum, the spore suspension of the fungus (1–2 × 10^6^ cells/mL) was inoculated using Czapek’s liquid medium with 2% glucose and incubated on a Certomat BS-1 shaker (230 rpm and 25 ± 1 °C). Crude MC-LR extract was introduced as an aqueous solution into the nutrient medium at concentrations of 0.1 μg/mL and 0.45 μg/mL. An abiotic control (no culture) was used to assess the loss of MC-LR under abiotic conditions. The growth of the fungal biomass was determined by the gravimetric method. To determine the degree of adsorption of microcystin by fungal cells, 250 mg (dw) of the inactivated fungal biomass was added to Czapek’s liquid medium with 2% glucose and 0.1 µg/mL MC-LR. Czapek’s medium; MC-LR without fungus biomass was used as a control. The flasks were incubated on a Certomat BS-1 shaker at 230 rpm and 25 ± 1 °C for 3 days. The supernatant was separated by centrifugation at 10,000 rpm for 5 min, and the content of MC-LR was determined.

### 4.4. Determination of the Algicidal Activity Spectrum of the Strain GF3

Green algae strains: *Oocystis parva* W. and G.S. West (CALU 391), *Scenedesmus quadricauda* (Turp.) Breb. (CALU 1248) and cyanobacteria: *Microcystis aeruginosa* Kütz. (CALU 973); *Planktothrix (Oscillatoria) agardhii* Gom Anagnostidis et Komarek (CALU 1113); *Aphanizomenon flos-aquae* (Linnaeus) Ralfs ex Bornet and Flahault (CALU 1033) obtained from the Resource Center “Cultivation of Microorganisms” of the Science Park of St. Petersburg State University (Russia); and *Anabaena cylindrica* Lemm. (HPDP) from the Institute of Hydrobiology of the National Academy of Sciences of Ukraine were used as test cultures.

The cyanobacteria and algae were grown under static conditions in 250 mL Erlenmeyer flasks containing 100 mL of BG11 medium [52]. Cultivation was carried out at illumination of 90 mol photons/m^2^/s, under a light/dark light mode of 12 h:12 h for 14 days at 25 °C. The cultures of the logarithmic phase of growth under the above conditions were used as inocula. The growth of test cultures was controlled by the content of chlorophyll *a*.

Chlorophyll *a* was extracted from the biomass with 90% acetone at 4 °C for 24 h. The optical densities of the acetone extract of chlorophyll *a* at wavelengths of 664, 647, and 630 nm were determined on a Genesys 10UV scanning spectrophotometer (Thermo electron corporation, Waltham, MA, USA). The chlorophyll *a* concentration (mg/L) was calculated using Equation (1) [53]:

chlorophyll *a* = 11.85 × A664 − 1.54 × A647 − 0.08 × A630, (1)

where A664, A647, and A630 are optical densities of the acetone extract of chlorophyll *a* at wavelengths of 664, 647, and 630 nm, respectively. 

To determine the algicidal activity of the GF3 strain, the 7-day culture liquid of the fungus grown on Sabouraud medium was centrifuged at 6000 rpm for 10 min. The supernatant was filtered through microfiltration membranes with a pore size of 0.22 μm (MFAS type, Vladipor, Vladimir, Russia) to obtain a cell-free fungal filtrate GF3. Pellets of the GF3 strain (100 mg dw) were washed with sterile 0.9% saline three times, and then resuspended in fresh BG11 medium. Equal volumes (10% vol.) of the culture liquid, mycelial suspension, and GF3 filtrate were added to BG11 medium (100 mL) simultaneously with the addition of test culture inoculums (10% vol.). The test cultures were cultured under the above conditions.

The algicidal effects (AE, %) of the culture liquid, GF3 filtrate, and the washed cells of the isolated strain were calculated as follows (2):

AE = (Cc − Ct)/Cc × 100,(2)

where Cc and Ct are the concentrations of chlorophyll *a* in the control and treatment variants, respectively.

### 4.5. Preparation of Microcystin-LR Crude Extract 

Microcystin crude extract was obtained from the biomass of the toxic strain *Microcystis aeruginosa* CALU 973 grown for 14 days under the above conditions. The culture liquid was centrifuged at 6000 rpm for 15 min. The concentrated biomass was frozen at −28 °C for a day, and then thawed at 22 ± 1 °C. The freeze–thaw procedure was repeated three times. The biomass was then homogenized on a Tissue Lysser LT (QIAGEN, Hilden, Germany) with 5 mm steel beads. Methanol at 80% was added to the homogenate in a volume equal to the volume of the homogenate. The extraction was carried out on a Certomat rotary shaker at 100 rpm for 1 h. Then, the extract was separated using centrifugation at 4 °C (6000 rpm, 10 min) and evaporated on a rotary evaporator IR-1M3 (Khimlaborpribor, Klin, Russia) at 40 °C. The crude extract was diluted with distilled water (10 mL) and stored at −28 °C. The concentration of MC-LR was 74.2 μg/mL in the crude extract.

### 4.6. Quantification of MC-LR and Identification of Its Transformation Products by LC–MS/MS Analysis

To determine the content of MC-LR and its transformation products in the native solution, the culture liquid of the fungus was centrifuged at 6000 rpm for 10 min. The aliquot of the native solution (5 mL) was subjected to solid-phase extraction using Oasis HLB cartridges (60 mg, Waters, Milford, MA, USA). After loading the sample, the cartridges were washed with 1 mL of deionized water, and the target compounds were eluted with 5 mL of methanol.

The quantification of MC-LR and the identification of its transformation products were carried out using high-performance liquid chromatography—high-resolution mass spectrometry using an LTQ Orbitrap XL mass spectrometer (30000) (Thermo Fisher Scientific, San Jose, CA, USA) with a Prominence LC-20 series liquid chromatograph (Shimadzu, Tokyo, Japan). The extract components were separated using a Luna Omega C18 column, 100 × 2.1 mm, 3 µm (Phenomenex, Torrance, CA, USA) in the gradient elution mode. The mobile phase consisted of water and acetonitrile with the addition of 0.05% formic acid. The column temperature was 40 °C [54].

The target compounds were identified according to the established retention times (with an error not exceeding 0.3 min), the exact *m*/*z* values of [M + H]^+^ and [M + 2H]^2+^ ions (the mass determination accuracy was 5 ppm) [55], and the data of complete fragment spectra [46]. The quantitative determination was carried out using the external standard method.

### 4.7. Assay of Antioxidants and Lipid Peroxidation

The superoxide dismutase (SOD) activity was determined by the ability of the enzyme to inhibit the photochemical reduction of nitroblue tetrazolium, according to the method of Beyer and Fridovich [56]. The absorbance was measured at 560 nm on a Genesys 10 UV scanning spectrophotometer (Thermo Spectronic, New York, NY, USA). The results were expressed in U/g dw. The catalase activity (CAT) was assessed spectrophotometrically using a Genesys 10 UV scanning spectrophotometer by the method in [57], and was expressed in U/g dw/min. To determine the content of reduced glutathione (GLU), a modified method of Gao and Tam was used [58]. The concentration of glutathione was determined from the calibration curve and expressed in nmol/g dw. To determine the content of malondialdehyde (MDA), the method presented by Dhindsa et al. [59] was used. The concentration of MDA was calculated, taking into account the extinction coefficient of 155 mM^−1^ cm^−1^ [60]. The results were expressed in µmol/g dw. Detailed descriptions of the procedures for determining SOD, CAT, GLU, and MDA were presented earlier [50,61].

### 4.8. Toxicity Analysis

The levels of toxicity of Chapek’s medium containing MC-LR (abiotic control) and GF3 filtrate were assessed using a biotest—a commercial test kit Daphtoxkit F (MicroBioTest Inc., Nazareth, Belgium) for acute toxicity [62,63]. *Daphnia magna* was used as a bioindicator of toxicity. Three independent analyses were performed in triplicate. The test results were expressed in toxicity units (TU) using Formula (3):

TU = 1/LC_50_ ×100, (3)

where LC_50_ (%)—GF3 filtrate concentration leading to 50% death of the tested bioindicators after 72 h. Lethal concentrations were determined from the linear part of the curve using regression analysis.

### 4.9. Statistical Analysis

Statistical analysis and graphical presentation of the results were carried out using Microsoft Excel 2007 and Past 4.0 software. The statistical significance of differences between the variants was determined using one-way ANOVA followed by the use of the Mann–Whitney U-test (*p* < 0.05). The data were presented as the arithmetic mean ± standard deviation (SD) of three independent biological replicates.

## Figures and Tables

**Table 1:** The detected MC congeners and their concentrations in the water sample collected in the Gulf of Finland in June 2018.

Microcystin Congeners	[M + H]^+^	MC Content, ng/L
[D-Asp^3^]MC-LR	981.541	36.3
MC-LR	995.5565	306.4
[D-Glu-OCH_3_^6^]MC-LR	1009.572	3.4
[Dha^7^]MC-HphR	1029.541	29.9
MC-YR	1045.536	66.7
Total MC content		442.7

## Data Availability

Not applicable.
